# Mortality, Severe Acute Respiratory Infection, and Influenza-Like Illness Associated with Influenza A(H1N1)pdm09 in Argentina, 2009

**DOI:** 10.1371/journal.pone.0047540

**Published:** 2012-10-31

**Authors:** Eduardo Azziz-Baumgartner, Ana María Cabrera, Loretta Chang, Rogelio Calli, Gabriela Kusznierz, Clarisa Baez, Pablo Yedlin, Ana María Zamora, Romina Cuezzo, Elena Beatriz Sarrouf, Andrea Uboldi, Juan Herrmann, Elsa Zerbini, Osvaldo Uez, Pedro Osvaldo Rico Cordeiro, Pollyanna Chavez, George Han, Julián Antman, Fatima Coronado, Joseph Bresee, Marina Kosacoff, Marc-Alain Widdowson, Horacio Echenique

**Affiliations:** 1 Influenza Division, U.S. Centers for Disease Control and Prevention, Atlanta, Georgia, United States of America; 2 Dirección de Epidemiología, Ministerio de Salud de la Nación, Buenos Aires, Argentina; 3 Epidemic Intelligence Service, U.S. Centers for Disease Control and Prevention, Atlanta, Georgia, United States of America; 4 Dirección de Epidemiología, Ministerio de Salud Pública Provincia de Tucumán, Tucumán, Argentina; 5 Instituto Nacional de Enfermedades Respiratorias, Santa Fe, Argentina; 6 Ministerio de Salud de la Provincia de Buenos Aires, Buenos Aires, Argentina; 7 Ministerio de Salud Pública de la Provincia de Tucumán, Tucumán, Argentina; 8 Promoción y Protección de la Salud, Ministerio de Salud Pública Santa Fe, Santa Fe, Argentina; 9 Servicio Virología - Instituto Nacional de Epidemiología “Dr. Juan H. Jara”, Mar de Plata, Argentina; 10 Organización Panamericana de la Salud, Buenos Aires, Argentina; National Institutes of Health, United States of America

## Abstract

**Introduction:**

While there is much information about the burden of influenza A(H1N1)pdm09 in North America, little data exist on its burden in South America.

**Methods:**

During April to December 2009, we actively searched for persons with severe acute respiratory infection and influenza-like illness (ILI) in three sentinel cities. A proportion of case-patients provided swabs for influenza testing. We estimated the number of case-patients that would have tested positive for influenza by multiplying the number of untested case-patients by the proportion who tested positive. We estimated rates by dividing the estimated number of case-patients by the census population after adjusting for the proportion of case-patients with missing illness onset information and ILI case-patients who visited physicians multiple times for one illness event.

**Results:**

We estimated that the influenza A(H1N1)pdm09 mortality rate per 100,000 person-years (py) ranged from 1.5 among persons aged 5–44 years to 5.6 among persons aged ≥65 years. A(H1N1)pdm09 hospitalization rates per 100,000 py ranged between 26.9 among children aged <5 years to 41.8 among persons aged ≥65 years. Influenza A(H1N1)pdm09 ILI rates per 100 py ranged between 1.6 among children aged <5 to 17.1 among persons aged 45–64 years. While 9 (53%) of 17 influenza A(H1N1)pdm09 decedents with available data had obesity and 7 (17%) of 40 had diabetes, less than 4% of surviving influenza A(H1N1)pdm09 case-patients had these pre-existing conditions (p≤0.001).

**Conclusion:**

Influenza A(H1N1)pdm09 caused a similar burden of disease in Argentina as in other countries. Such disease burden suggests the potential value of timely influenza vaccinations.

## Introduction

Early during the 2009 pandemic, the number of deaths attributed to influenza A(H1N1)pdm09 in Argentina (population 40 million [1]) was only surpassed by the number of deaths in United States (population 300 million [2]). A case-series conducted during the first months of the pandemic suggested that influenza A(H1N1)pdm09 mortality rates were at least 0.5–1.1/100.000 and concentrated among middle-aged adults with comorbidities [3]. The case-fatality proportion among hospitalized case-patients was 18% during June, 2009 [3]. Such findings suggested that the burden of influenza A(H1N1)pdm09 in Argentina was greater than that elsewhere.

A subsequent review of surveillance data suggested that the apparent influenza-associated disease burden in Argentina was likely an artifact of the way that the initial severe case patients were preferentially sampled and reported to authorities. Indeed subsequent surveillance data from Argentina suggested that the proportion of persons infected with influenza A(H1N1)pdm09 who died as a result of their illness may have been similar to that elsewhere. In order to explore whether Argentina's influenza A(H1N1)pdm09 burden was higher or similar to the burden documented elsewhere, we use active facility-based influenza surveillance and health utilization surveys from three cities in Argentina to estimate rates of influenza A(H1N1)pdm09-associated mortality, hospitalization, and influenza-like illnesses.

## Methods

### Study population

This study was conducted in three cities in Argentina, Mar de Plata (central Argentina, population 701,096), Tucumán (the largest city in northern Argentina, population 1,493,488) , and Santa Fe (east-central Argentina, population 396,243) that comprised 6% of the total population of 40 million persons in Argentina. During April–December 2009 (epidemiological weeks 14–52), surveillance staff actively searched city and hospital ledgers for all decedents with a history, in the previous two-weeks, of influenza-like illness (ILI), defined as persons with sudden onset fever [≥38°C], with cough or sore throat [4]. In addition, surveillance staff actively searched for all persons hospitalized with severe acute respiratory infection (SARI), defined as persons with sudden onset fever [≥38°C] with cough or sore throat requiring hospitalization as a result of complications from ILI [4]. Last, because of limited personnel, surveillance staff actively searched for a convenience sample of ILI case-patients who sought care among all sentinel city providers. Clinicians in these sentinel cities were also mandated to report all ILI case-patients whom they tested for influenza to the national surveillance system.

### Active surveillance case-patient questionnaires and laboratory sampling

For each SARI and ILI case patient, staff recorded the person's age; sex; date of illness onset; history of asthma or chronic obstructive pulmonary disease, diabetes, obesity, pregnancy; and survival status. As part of clinical care, a proportion of these case-patients provided both nasal and throat swabs for influenza testing during the course of their acute illness [4]. These were then tested by indirect immunofluorescence (sensitivity of ∼95%) [5] and/or reverse transcription-polymerase chain reaction to identify influenza type A and B, universal swine (primers and probes designed to identify swine influenzas), and 2009 H1N1 at one of the National Influenza Centers in Argentina using methods previously described (sensitivity of ∼98%) [4], [6], [7].

### Health utilization surveys

We conducted door to door cross-sectional surveys in each of the sentinel city populations during May–November 2010, the seasonal influenza epidemic months in Argentina, to determine if household members had developed ILI, if they had sought care, and the proportion who visited a physician multiple times for a single illness event (and therefore generated multiple case-reports in the national surveillance system). We assumed that the proportion of case-patients visiting physicians multiple times were similar during 2009 and 2010 and used bootstrapping to determine the 95% confidence interval for this proportion. We also estimated the proportion of persons in the population who were pregnant, obese, diabetic, asthmatic, or had chronic obstructive pulmonary disease and developed ILI.

### Estimating burden of disease by age-group

For each case-patient (decedent with a history of ILI, SARI, and ILI-case-patient) of a particular age group, we estimated the number of case-patients associated with influenza A(H1N1)pdm09 illness each week by adding case-patients which tested positive for influenza A(H1N1)pdm09 to the number of untested case-patients who may have tested positive for influenza A(H1N1)pdm09 if a respiratory sample had been obtained (Appendix S1). We obtained this number by multiplying the number of untested case-patients identified each week by the proportion of case-patients of the same age group which tested positive for influenza A(H1N1)pdm09 and this proportion's 95% confidence interval (Appendix S1). We calculated the number of ILI associated with influenza A(H1N1)pdm09 by adding the number ILI-case patients which tested positive for influenza A(H1N1)pdm09 to the number of untested ILI cases reported to the obligatory surveillance system multiplied by the proportion of ILI cases testing positive for influenza A(H1N1)pdm09 identified through active surveillance each week and this proportion's 95% confidence interval (Appendix S1).

We adjusted each numerator by the proportion of persons without information about their date of illness onset (i.e. epidemiologic week of illness) and, in the case of ILI case-patients, for the proportion of persons who sought care multiple times per illness event as estimated by the health utilization surveys (Appendix S1). Next, we estimated the rate of influenza A(H1N1)pdm09 -associated case-patients by dividing the sum of influenza-associated decedents, SARI-case patients, and ILI-case-patients by the age-specific census population in the sentinel sites catchment during 2009 (Appendix S1) [1]. Last, we compared the probability that decedents with a history of ILI, SARI, and ILI case-patients were more likely to be of a certain age or have a pre-exiting medical condition using rank-sum tests and Fisher's Exact tests.

### Ethical aspects

The research protocol was reviewed and approved by the Argentina Ministry of Health and the Argentina office of the Pan American Health Organization. Health authorities strived to maintain case-patient confidentiality by protecting data elements that help third parties identify them. Health utilization survey participants provided verbal informed consent prior to participation.

## Results

### Rates of *influenza A(H1N1)pdm09* -associated mortality

**Figure 1:**
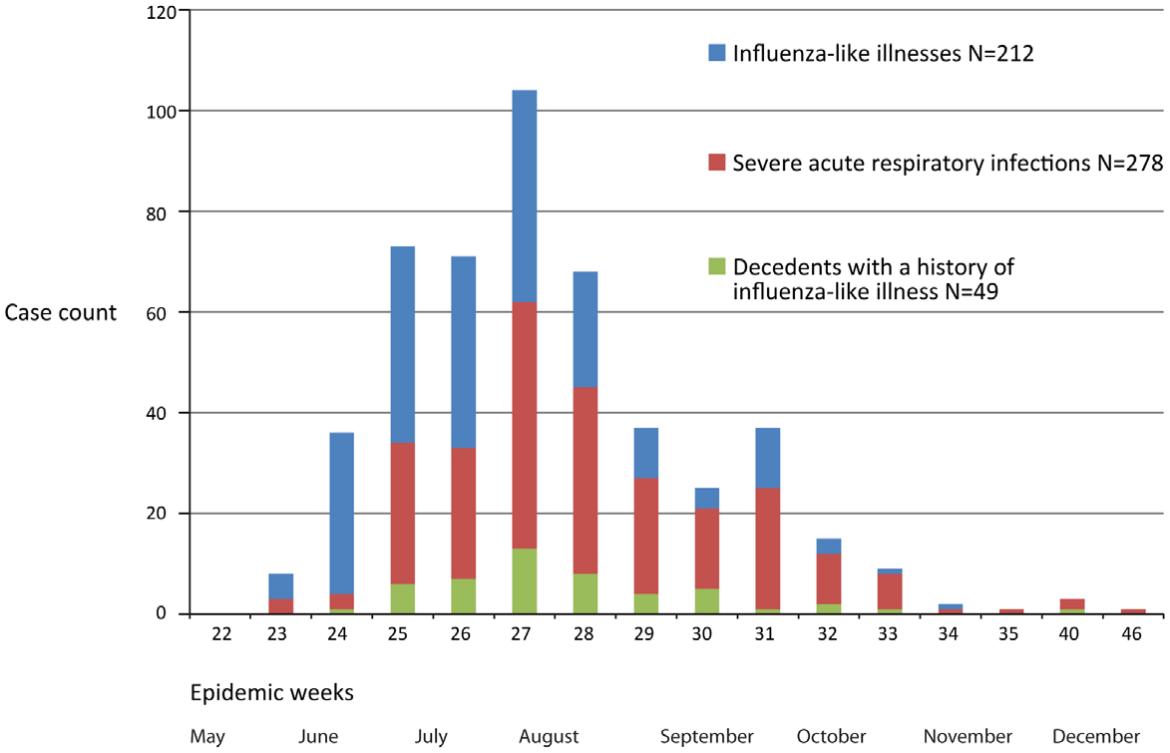
Distribution of influenza A(H1N1)pdm09-associated case-patients in three cities in Argentina by epidemiologic week, May–December 2009.

Investigators identified 108 decedents with a history of ILI during the study period (Table 1) (Figure 1). Sixty-five (61%) of 108 were males. The median age of the 74 decedents tested for influenza was 50 years compared to 61 years among the 34 untested decedents (p = 0.01). Laboratorians identified influenza A(H1N1)pdm09 among 49 (66%) of 74 decedents tested for influenza. Accounting for age and epidemiologic week, we estimated that 66 persons died with influenza A(H1N1)pdm09 illness within our study population. We divided this estimate by the age-appropriate census population, adjusted for the proportion of decedents without known symptom onset (4 [4%] of 108), and estimated that the influenza A(H1N1)pdm09-associated mortality rate per 100,000 ranged from 1.5 (95%CI 1.5–1.7) among persons aged 5–44 years to 5.6 (95%CI 5.6–5.6) among persons aged ≥65 years.

**Table 1:** Rates of influenza A(H1N1)pdm09-associated mortality among influenza-like illness (ILI) cases at three sentinel cities in Argentina, according to age group. April–December, 2009.

Row number, Category	Age group				
	<5 years	5–44 years	45–64 years	≥65 years	All ages
1. Number of decedents with a history of ILI identified through active surveillance with date of illness onset information	6	34	39	25	104
2. Influenza A(H1N1)pdm09 positives among those sampled, n/N (%)	4/4 (100%)	22/30 (73%)	15/27 (56%)	8/13 (62%)	49/74 (66%)
3. Estimated number of influenza A(H1N1)pdm09-associated deaths^a^	4 (95%CI 4–4)	24 (95%CI 26–24)	19 (95%CI 21–18)	13 (95%CI 13–13)	66 (95%CI 72–59)
4. Population estimates for area under active surveillance	222,681	1,549,706	489,965	250,019	2,512,370
5. Estimated rates of 2009 H1N1mortality per 100,000 persons^b^	1.8 (95%CI 1.8–1.8)	1.5 (95%CI 1.7–1.5)	4.1 (95%CI 4.4–3.8)	5.6 (95%CI 5.6 –5.6)	2.7 (95%CI 3.0–2.4)

a Estimated by multiplying the week-specific number of decedents identified (row 1) by the week-specific proportion testing positive for 2009 H1N1 (row 2) and its 95% confidence interval.

b Corrected for the proportion of case-patients missing date of illness onset (epidemiologic week) information (i.e. 2 of 39 among persons aged 45–64 years and 2 of 27 among persons aged ≥65 years).

### Rates of 2009 H1N1-associated SARI

Investigators identified 1,622 SARI case-patients (Table 2) (Figure 1). Eight-hundred and twenty-two (51%) were males. Among those with available age information, the median age of the 687 SARI case-patients tested for influenza A(H1N1)pdm09 was 27 years compared to 36 years among the 887 untested SARI case-patients (p<0.0001). Laboratorians identified influenza A(H1N1)pdm09 among 279 (45%) of 621 SARI case-patients with adequate laboratory samples. After accounting for age and epidemiologic week, we estimated that 600 persons of all ages developed SARI associated with influenza A(H1N1)pdm09. We divided these estimates by the age-appropriate census population and estimated that the influenza A(H1N1)pdm09-associated SARI rate per 100,000 ranged from 26.9 (95%CI 35.4–18.4) among persons aged <5 years to 41.8/100,000 (95%CI 66.5–18.3) among persons aged ≥65 years.

**Table 2:** Rates of influenza A(H1N1)pdm09-associated severe acute respiratory case-patients at three sentinel cities in Argentina, according to age group May–December, 2009.

Row number, Category	Age Group				
	<5 years	5–44 years	45–64 years	≥65 years	All ages
1. Number of SARI hospitalizations identified through active surveillance with date of illness onset information	270	741	344	252	1,607
2. Influenza A(H1N1)pdm09 positives among those sampled, n/N (%)	32/143 (22%)	154/345 (45%)	64/144 (44%)	28/67 (42%)	279/621 (45%)
3. Estimated number of influenza A(H1N1)pdm09-associated severe acute respiratory infections^a^	60 (95% CI 78–41)	319 (95% CI 376–262)	134 (95% CI 173–96)	104 (95%CI 166–45)	600 (95%CI 698–504)
4. Population projections by age group for area under active surveillance during 2009	222,681	1,549,706	489,965	250,019	2,512,370
5. Estimated rates of 2009 H1N1SARI hospitalizations per 100,000 persons^b^	26.9 (95% CI 35.4–18.4)	20.8 (95%CI 24.5–17.1)	27.9 (95% CI 35.8–19.9)	41.8 (95% CI 66.5–18.3)	24.1 (95% CI 28.0–20.2)

a Estimated by multiplying the week-specific number of decedents identified (row 1) by the week-specific proportion testing positive for 2009 H1N1 (row 2) and its 95% confidence interval.

b Corrected for the proportion of case-patients missing date of illness onset (epidemiologic week) information (i.e. 1 of 271 among children aged <5 years, 7 of 748 among persons aged 5–44, 6 of 350 among persons aged 45–64 years, and 1 of 253 among persons aged ≥65 years).

### ILI case patients identified through obligatory and active surveillance

Clinicians at the three sentinel cities reported 101,179 ILI cases to the national obligatory surveillance system when physicians submitted samples for respiratory virus testing (Table 3). Investigators at the sentinel cities also identified through active surveillance 22,474 ILI case-patients who sought medical care (Figure 1). Approximately half (10,815 [48%]) of the 22,465 patients with available information were male. The median age of the 989 ILI case-patients tested for influenza A(H1N1)pdm09 was 8 years compared to 22 years among the 20,803 untested ILI case-patients (p<0.0001). Laboratorians identified influenza A(H1N1)pdm09 among 212 (21%) of 996 ILI case-patients with laboratory samples.

**Table 3:** Rates of influenza A(H1N1)pdm09-associated influenza-like illness case-patients at three sentinel cities in Argentina, according to age group April–December, 2009.

Number, Category	Age Group				
	<5 years	5–44 years	45–64 years	≥65 years	All ages
1. Number of ILI case patients reported to the national influenza surveillance system from sentinel cities when their physicians ordered an influenza laboratory test with date of illness onset information	17,302	68,968	11,880	3,209	101,179
2. Number (%) of ILI case-patients with respiratory samples of all identified through active surveillance with date of illness onset information	405/2,268 (18%)	490/15,514 (3%)	73/2,624 (3%)	28/1,117 (3%)	996/21,523 (5%)
3. Influenza A(H1N1)pdm09 positives among those sampled, n/N (%)	27/405 (7%)	157/490 (32%)	24/73 (33%)	4/28 (14%)	212/996 (21%)
4. ILI events among total visits to physicians among residents surveyed , n/(%)	124/224 (55%)	286/437 (65%)	77/113 (68%)	30/46 (65%)	517/820 (63%)
5. Estimated number of influenza A(H1N1)pdm09-associated influenza-like illnesses^a^	5,996 (95% CI 12,433–1,272)	339,969 (95% CI 559,282–139,395)	118,312 (95% CI 42.8–1.7)	25,761 (95% CI 28,239–24,212)	283,783 (95% CI 421,897–151,882)
6. Population projections by age group for three cities under active surveillance during 2009	222,681	1,549,706	489,965	250,019	2,512,370
7. Rates of 2009 H1N1among health-care seeking ILI case-patients per 100 persons by age group (95% CI)^b^	1.6 (95% CI 3.8–0.3)	15.0 (95% CI 26.6–5.7)	17.1 (95% CI 42.8–1.7)	7.1 (95% CI 9.5–5.2)	7.4 (95% CI 11.8–3.8)

a Estimated by multiplying the week-specific number of ILI cases identified (row 1) by the proportion testing positive for 2009 H1N1 (row 3) and its 95% confidence interval by the proportion of ILI cases among ILI physician visits (row 4) while adjusting for the proportion of cases-patients missing data on their epidemiologic week of illness.

b Corrected for the proportion of case-patients missing date of illness onset (epidemiologic week) information (i.e. 95 of 2,365 among children aged <5 years, 688 of 16,204 among persons aged 5–44, 101 of 2725 among persons aged 45–64 years, and 63 of 1180 among persons aged ≥65 years).

### Health utilization surveys

Staff interviewed 14,535 households with 22,066 household members (mean 1.5 persons per household). Seven hundred fifty-two (3.4%) of the 22,066 household members reported a history of ILI during the month before the interview. The ILI case patients had a median age of 20 years (IQR 7–40 years) and 339 (45%) of 752 were male (Table 4). Of ILI case-patients with available data, 50 had asthma, 47 had chronic obstructive pulmonary disease, 22 had diabetes, and 13 had obesity. Nine of 164 women aged 15–50 were pregnant. Among 517 case-patients with ILI, 336 (65%) sought care from a physician one time, 120 (23%) two times, 36 (7%) three times, 17 3%) four times, 2 (0.4%) five, 5 (1%) six times, 4 (0.8) seven times for the same illness. Therefore, we estimate that the 517 ILI case-patients generated 820 physician visits and that there were approximately 0.63 illness events per patient visit [95% confidence interval (CI) 0.6–0.7]).

**Table 4:** Description of case-patients with available risk factor data identified during active case-finding in 2009 and during three consecutive health utilization surveys in 2010, three cities in Argentina.

Characteristic^a^	Decedents with a history of influenza and respiratory samples positive for influenza A(H1N1)pdm09. N = 49	SARI case-patients with respiratory samples positive for influenza A(H1N1)pdm09. N = 278	Health-care seeking ILI case-patients with respiratory samples positive for influenza A(H1N1)pdm09 with available information. N = 209	General population in the sentinel sites surveyed with available data (95% confidence interval). N = 752 ILI case-patients
Median age (interquartile range)^b^	37 (19–57)	30 (16–51)	19 (10–34	20 (7–40)
Females, n/N (%)^c^	22/49 (45%)	136/231(41%)	104/212 (49%)	409/748 (51.1–58.3%)
Pregnancy among women aged 15–50 years, n/N (%)^d^	1/6 (17%)	34/66 (51%)	3/33 (9%)	9/164 (2–9%)
Obesity, n/(%)^d^	9/17 (53%)	12/92 (13%)	0/29 (0%)	13/738 (0.8–2%)
Diabetes n/N (%)^d^	2/29 (7%)	9/167 (5%)	2/114 (2%)	22/740 (1.7–4.2%)
Chronic obstructive pulmonary disease n/N (%)^d^	1/29 (3%)	2/167 (1%)	0/114 (0%)	47/742 (4.6–8.1)
Asthma n/N (%)^d^	1/15 (6%)	5/90 (5%)	2/32 (6%)	50/740 (4.9–8.6)

2009 H1N1 denotes influenza A (H1N1)pdm09, SARI denotes severe acute respiratory illness, and ILI denotes influenza-like illness.

a among cases with available age, pregnancy, obesity, chronic obstructive pulmonary disease, or asthma status data.

b The age of case-patients is significantly associated with decedent, SARI, or ILI case-status in linear regression (p≤0.001).

c The sex of case-patients is significantly associated with decedent, SARI, or ILI case-status in Fisher Exact testing (p = 0.01).

d The characteristic is significantly associated with decedent, SARI, or ILI case-status in Fisher Exact testing (p<0.001).

### Rates of 2009 H1N1-associated ILI

We estimated that the influenza A(H1N1)pdm09-associated ILI rates per 100 py were 1.6 (95% CI 3.8–0.32) among children aged <5 years, 15.0 (95% CI 26.6–5.7) among persons aged 5–44 years, 17.1 (95% CI 42.8–1.7) among persons aged 45–64, and 7.1 (95% CI 9.5–5.2) among persons aged ≥65 years (Table 3).

### Risk factors for death among health seeking 2009 H1N1 case-patients

Decedents were more likely than other case-patients to be pregnant (2 [25%] of 8 vs. 667 [5%] of 112,910 among women aged 15–50 years, p = 0.01), obese (13 [35%] of 37 vs. 69 [0.4%] of 18,994, p<0.001), or diabetic (9 [17%] of 52 vs. 983 [2%] of 46,888, p<0.001). While 9 (53%) of 17 influenza A(H1N1)pdm09 decedents with available data had obesity and 7 (17%) of 40 had diabetes, less than 4% of surviving influenza A(H1N1)pdm09 case-patients had these pre-existing conditions (p≤0.001).

## Discussion

Our findings suggest that influenza A(H1N1)pdm09 caused a significant burden of disease in Argentina during 2009. If we assume that influenza activity was similar throughout country, we could multiply the age specific influenza-associated rates from Santa Fe, Tucumán, and Mar del Plata and their 95% confidence intervals by the census population of Argentina (3,240,001 persons aged <5 years; 25,055,187 aged 5–44 years; 7,717,549 aged 45–64; and 4,121,684 aged ≥65 years) to estimate that approximately 1,300,000 (95% CI 10,000,000–1,800,000) persons visited clinicians for ILI, 10,000 (95% CI 13,000–7,200) were hospitalized, and 990 died (95% CI 1,100–970) throughout the country as a result of the 2009 pandemic.

Our mortality pandemic rates were more conservative than those estimated using linear models of 2009 Argentina pneumonia and influenza mortality data (8.4/100,000py (95% CI 6.5–10.3/100,000py) a method that is not well suited to differentiate the impact of influenza from that or RSV [9]. Our estimates are similar, however, to age-adjusted influenza A(H1N1)pdm09-associated respiratory and cardiovascular rates for the southern cone countries (2.1–4.0/100,000py) [8]. Indeed, our estimates were similar to those of diverse countries such as Bangladesh (4/100,000py) [9] and subpopulations within the United States (0.9–3.7/100,000py) [9], [10].

Influenza A(H1N1)pdm09 mortality and hospitalization rates were also similar to those of seasonal influenza [11], [12], [13]. Influenza A(H1N1)pdm09 mortality was similar to Argentina's seasonal influenza mortality estimated using pneumonia and influenza diagnostic codes and Serfling models (2.3–10.6/100,000 person-years during 2002–2009) [9] and auto-regressive integrated moving averages models (0–4.6/100,000py during 1992–2002) [11], [12]. Our findings, therefore, suggest that early case-fatality proportions overestimated the actual burden of influenza A(H1N1)pdm09. Plausible explanations for this early overestimation include clinicians' preferentially identifying, sampling, and reporting severely ill case-patients. Nevertheless, it is important to note that while the overall influenza rates may be have been similar during 2009 and previous years, the years to life lost were likely greater during the pandemic because a greater proportion of ill persons were aged <65 years than during a typical influenza season.

### Estimates of national influenza burden

Influenza A(H1N1)pdm09-associated hospitalizations were similar to those reported in Australia during 2009 (23/100,000py) [14] but higher to those of low income countries such as Bangladesh (13/100,000py hospitalizations) [15]. The similarities between Argentina's medically attended ILI rates and those of Bangladesh (6.6/100py) [15], however, suggests that differences in hospitalization rates may be a factor of health utilization.). As with influenza mortality, Argentina's pandemic and seasonal influenza hospitalization rates were similar (20/100,000 influenza-associated pneumonia and influenza hospitalizations and 60/100,000 influenza-associated respiratory and cardiac hospitalizations during 2005–2008 [13]).

A higher proportion of influenza decedents were pregnant, obese, or diabetic when compared with surviving ILI case-patients [3] [16]. Such findings suggest that ministries of health may be justified in exploring the burden of seasonal influenza in these groups and whether measures used to control and prevent influenza during the pandemic [17] would be applicable to prevent and mitigate disease among subpopulations at high risk of complications from seasonal influenza illness [18].

Our study found that the proportion of decedents with positive 2009 H1N1 samples (66%) was significantly higher than that of hospitalized SARI case-patients (42%) and ILI case-patients (25%). Such a finding suggests that while influenza was identified in a fraction of ILI case-patients [15] and community acquired pneumonias [19], [20], influenza can be identified among a significant proportion (1/3–2/3) of severe hospitalized illness case-patients and decedents in during epidemic periods. If consistent among other surveillance platforms, such findings could have implications for countries exploring whether to empirically treat SARI case-patients with oseltamivir or other antivirals during influenza epidemic periods [21].

### Limitations

This study had several important limitations. We assumed that after accounting for case-definition, age-group, and epidemiologic week, the proportion of tested and untested case-patients with influenza A(H1N1)pdm09 was likely similar. This may be incorrect if physicians were more likely to test severely ill younger case-patients without pre-existing medical conditions, if the laboratory used one assay preferentially to test severely ill case-patients, and if the probability of testing positive for influenza A(H1N1)pdm09 was greater among severely ill case-patients. Although improbable, it is mathematically feasible that all un-tested case-patients had influenza A(H1N1)pdm09 infection, (a theoretical scenario where our influenza A(H1N1)pdm09-associated ILI rates would have been 50/100py, the hospitalization rates 48/100,000py, and the mortality rates 3.3/100,000py). Conversely, all un-tested case-patients may have been infected with other pathogens and not influenza (a theoretical scenario where our influenza A(H1N1)pdm09-associated ILI rates would have been 1/100py, the hospitalization rates 11/100,000py, and the mortality rates 2.0/100,000py). Last, we assumed that proportion of persons seeking care multiple times for ILI was similar during the 2010 and 2009 epidemic periods.

## Conclusions

Our study suggests that influenza A(H1N1)pdm09 burden in Argentina was similar to that elsewhere and caused a large number of deaths, hospitalizations, and cases of ILI. Indeed, the majority of decedents with a history of ILI identified during the pandemic tested positive for influenza A(H1N1)pdm09. Influenza-associated mortality and hospitalization rates were similar to those elsewhere and to those of Argentina during seasonal influenza epidemics. Such findings suggest that it may be prudent to examine interventions used during the pandemic to determine their potential value to prevent and mitigate Argentina's annual seasonal influenza burden.

## Supporting Information

Appendix S1 **Equation for calculating the rates of influenza A(H1N1)pdm09-associated mortality among decedents with a history of influenza-like illness (ILI) at three sentinel cities**
(DOC)
