# Supplementary material for: Mortality, Severe Acute Respiratory Infection, and Influenza-Like Illness Associated with Influenza A(H1N1)pdm09 in Argentina, 2009
Source: PLoS One. 2012 Oct 31;7(10):e47540. doi: 10.1371/journal.pone.0047540 (PMC3485247; doi:10.1371/journal.pone.0047540)
Supplement: Appendix S1 — Equation for calculating the rates of influenza A(H1N1)pdm09-associated mortality among decedents with a history of influenza-like illness (ILI) at three sentinel cities (DOC) [file pone.0047540.s001.doc]

**Appendix**:

Equation for calculating the rates of influenza A(H1N1)pdm09-associated mortality among decedents with a history of influenza-like illness (ILI) at three sentinel cities


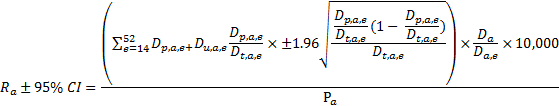


Where:

R_a_ = Rates of influenza A(H1N1)pdm09-associated mortality per 100,000 persons by age group

CI=confidence interval

e = epidemiologic week

D_p,a,e_ = Number of decedents with a history of ILI identified through active surveillance testing positive for influenza A(H1N1)pdm09 by age group and epidemiologic week

D_u,a,e_ = Number of decedents with a history of ILI identified through active surveillance untested for influenza A(H1N1)pdm09 by age group and epidemiologic week

D_t,a,e_ = Number of decedents with a history of ILI identified through active surveillance tested for influenza A(H1N1)pdm09 by age group and epidemiologic week

D_a_ = Number of decedents with a history of ILI identified through active surveillance by age group with or without illness-onset (i.e. epidemiologic week) information

D_a,e_ = Number of decedents with a history of ILI identified through active surveillance by age group with illness-onset (i.e. epidemiologic week) information

P_a_ = Population projections for area under active surveillance during 2009 by age group

Equation for calculating the rates of influenza A(H1N1)pdm09-associatedsevere acute respiratory infections (SARI) at three sentinel cities


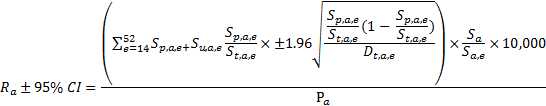


Where:

R_a_ = Rates of influenza A(H1N1)pdm09-associated severe acute respiratory case-patients (SARI) per 100,000 persons by age group

CI=confidence interval

e = epidemiologic week

S_p,a,e_ = Number of severe acute respiratory case-patients (SARI) identified through active surveillance testing positive for influenza A(H1N1)pdm09 by age group and epidemiologic week

S_u,a,e_ = Number of severe acute respiratory case-patients (SARI) identified through active surveillance untested for influenza A(H1N1)pdm09 by age group and epidemiologic week

S_t,a,e_ = Number of severe acute respiratory case-patients (SARI) identified through active surveillance tested for influenza A(H1N1)pdm09 by age group and epidemiologic week

S_a_ = Number of severe acute respiratory case-patients (SARI) identified through active surveillance by age group with or without illness-onset (i.e. epidemiologic week) information

S_a,e_ = Number of severe acute respiratory case-patients (SARI) identified through active surveillance by age group with illness-onset (i.e. epidemiologic week) information

P_a_ = Population projections for area under active surveillance during 2009 by age group


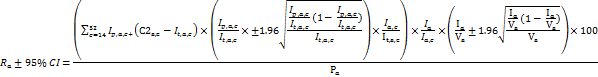


Where:

R_a_ = Rates of influenza A(H1N1)pdm09-associated influenza-like illness (ILI) per 100,000 persons by age group

CI=confidence interval

e = epidemiologic week

I_p,a,e_ = Number of influenza-like illness (ILI) identified through active surveillance testing positive for influenza A(H1N1)pdm09 by age group and epidemiologic week

C2_a_ = Number of ILI case patients reported to the national influenza surveillance system (aka. C2) from study cities when their physicians ordered an influenza laboratory test by age group and epidemiologic week

I_t,a,e_ = Number of influenza-like illness (ILI) identified through active surveillance tested for influenza A(H1N1)pdm09 by age group and epidemiologic week

I_a,e_ = Number of influenza-like illness (ILI) identified through active surveillance by age group and epidemiologic week with or without laboratory testing

I_a_ = Number of influenza-like illness (ILI) identified through active surveillance by age group with or without illness-onset (i.e. epidemiologic week) information

I_a_ = Number of ILI events identified through cross-sectional surveys of sentinel site catchment area residents by age group

V_a_ = Number of visits to physicians survey participants reported per illness event among all surveyed

P_a_ = Population projections for area under active surveillance during 2009 by age group
